# Comprehensive evaluation of the effect of five sterilization methods on the quality of black carrot juice based on PCA, TOPSIS and GRA models

**DOI:** 10.1016/j.fochx.2023.100604

**Published:** 2023-02-21

**Authors:** Shihan Bao, Dingze Yin, Qinyu Zhao, Yuan Zhou, Yayun Hu, Xiangyu Sun, Xuebo Liu, Tingting Ma

**Affiliations:** a College of Food Science and Engineering, College of Enology, Northwest A&F University, Yangling 712100, China; b College of Enology, Shaanxi Provincial Key Laboratory of Viti-Viniculture, Viti-viniculture Engineering Technology Center of State Forestry and Grassland Administration, Shaanxi Engineering Research Center for Viti-Viniculture, Heyang Viti-viniculture Station, Ningxia Eastern Foot of Helan Mountain Wine Station, Northwest A&F University, Yangling 712100, China

**Keywords:** Black carrot juice, Thermal sterilization, High hydrostatic pressure, Thermosonication, Comprehensive evaluation model

## Abstract

The effect of thermal pasteurization (TP), high temperature long time (HTLT), ultra-high temperature instantaneous (UHT), high hydrostatic pressure (HHP) and thermosonication (TS) sterilization on the physicochemical, sensory and functional properties of black carrot juice (BCJ) were studied. And for the first time, the comprehensive quality of sterilized BCJ was quantified by mathematical modeling. UHT was the least suitable sterilization method for BCJ resulting from the most severe deterioration in functional properties. TS had adverse effects on sensory and physicochemical properties, but significantly increased the total flavonoids and anthocyanins contents (*p* < 0.05) and showed the strongest antioxidant activity, making it a nutritional high-value processing method. TP and HHP balanced the improvement of sensory properties and the retention of functional properties, which were the most suitable sterilization methods for BCJ. This study determined the optimal sterilization methods of BCJ, and provided a scientific solution for the screening of high quality processing methods.

## Highlights

• The comprehensive quality of BCJ was quantitatively analyzed by mathematical modeling.
• Polyphenol and carotenoid are stable in BCJ, sterilization has little effect on them.
• TS is a nutritional high-value processing of BCJ, but causes sensory deterioration.
• TP and HHP are the most suitable sterilization methods for BCJ.
• Explore suitable sterilization process, not pursuing non-thermal technology blindly.

## Graphical abstract

**Figure:**
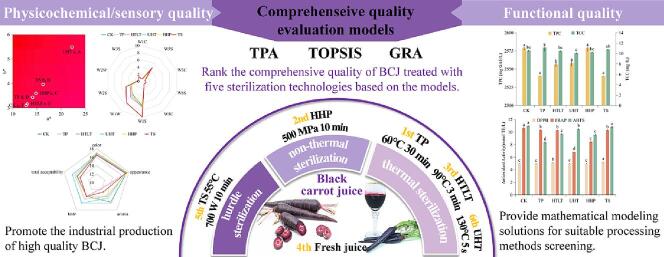

## Introduction

Black carrot (*Daucus carota* L.) is an excellent source of anthocyanins, containing up to 154 mg/g, of which 83% are acylated (Mizgier et al., 2016), and the acyl groups greatly enhance their stability under unfavorable conditions such as light, heat, and neutral and weakly acidic substrates (Zhao et al., 2017). Due to anthocyanins, the antioxidant activity of black carrot is 10–35 times that of other color carrots (Singh, Koley, Maurya, Singh, & Singh, 2018), with proven benefits for cancer, cardiovascular diseases and diabetes (Akhtar et al., 2017). Carrot juice is an ideal nutritional drink. Sterilization is the key step in its processing and is essential to control the safety, quality, stability and shelf life (Ma et al., 2022). At present, the sterilization of carrot juice is dominated by thermal sterilization, leading to quality issues including the loss of heat-sensitive nutrients, the deterioration of color and flavor. To break through the limitations of thermal sterilization, non-thermal technology such as high hydrostatic pressure (HHP), ultrasound and pulsed light were applied to carrot juice, and their effects on sensory, physicochemical, nutritional and functional properties were evaluated (Nadeem et al., 2018, Szczepańska et al., 2020, Zhu et al., 2019). Of all the non-thermal sterilization, HHP is the most developed and widely used at the industrial level (Ma et al., 2022). Additionally, hurdle sterilization is also an important strategy to avoid the disadvantages of thermal sterilization. Among them, thermosonication (TS) can inactivate microorganisms and enzymes at lower temperature and shorter treatment time due to the combination of mild heat and cavitation (Anaya-Esparza et al., 2017). Moreover, TS has no serious effects on juice quality, and may even increase the content of bioactive compounds, which has been evidenced as a promising sterilization technology of juice (Urango, Strieder, Silva, & Meireles, 2022). For carrot juice, TS can improve its quality by inhibiting enzymatic and microbial activity, reducing physicochemical changes and enhancing bioactive compounds (Jabbar et al., 2015, Martínez-Flores et al., 2015).

However, all the aforementioned studies only analyzed the effects of sterilization treatments on the sensory, physicochemical and nutritional indicators of carrot juice, and did not consider the role of these indicators in comprehensive quality judgment. Quality features as complex and comprehensive, including sensory, physicochemical, nutritional and functional aspects, thus a single type of indicators cannot evaluate the quality intuitively and accurately. Therefore, methods of using mathematics to establish the evaluation models play a crucial role in product comprehensive quality analysis. At present, comprehensive quality evaluation models based on Principal Component Analysis (PCA), Technique for Order Preference by Similarity to Ideal Solution (TOPSIS) and Gray Relational Analysis (GRA) have been applied to baijiu (Liu et al., 2022), tofu (Wang et al., 2021a), tea (Wang et al., 2021b), fruits and vegetables and their products (Khodaei et al., 2021, Liang et al., 2019), contributing greatly to processing-adapted variety screening, product grade classification and processing method optimization.

Although some studies evaluated the effect of sterilization methods on carrot juice quality, most of them were based on orange carrot juice, few studies on black carrot juice (BCJ), and there is a lack of comprehensive comparison of thermal, non-thermal and hurdle sterilization. Moreover, all the above studies were conducted with a single type of indexes, making it difficult to judge the comprehensive quality. Accordingly, this study investigated the effects of three thermal (thermal pasteurization TP, high temperature long time HTLT, ultra-high temperature instantaneous UHT), one non-thermal (HHP) and one hurdle (TS) sterilization on physicochemical, sensory and functional properties of BCJ. Besides, the evaluation models were established based on TOPSIS, GRA and PCA to quantify the comprehensive quality, so that it could be directly judged by the score. The research aims to clarify the optimal sterilization method of BCJ, and to explore the feasibility of evaluating the comprehensive quality of juice through mathematical modeling, so as to provide new solutions for the screening of high quality processing methods.

## Materials and methods

### Materials and chemicals

Black carrots “Purple 68” were purchased from TMALL. Gallic acid (GA), catechol (CA), cyanoside-3-glycoside (C3G), 6-hydroxy-2,5,7,8-tetramethylchromane-2-carboxylic acid (Trolox), Folin Ciocalteu, 1,1-diphenyl-2-picrylhydrazyl (DPPH), 2,6-dichloroindophenol,2,4,6-tripyridyl-s-triazine (TPTZ), and 2,2'-Azinobis-(3-ethylbenzthiazoline-6-sulphonate) (ABTS) were purchased from Sigma-Aldrich (St. Louis, MO, USA). Other reagents were purchased from Aladdin (Shanghai, China).

### BCJ preparation

The preparation of BCJ was performed according to the method described by Zhang et al. (2016). The carrots were washed, peeled, cut into slices and blanched for 5 min at 90℃ in 0.2% Vc (w/v) water solution. Then the slices were mixed with blanched water in a 1:1 (w:w) ratio and pulped until uniform. The pulp was filtered using 200-mesh gauze and centrifuged at 7000 *g* for 20 min at 4℃ (GL-10MD, Xiangyi, Hunan, China) to obtain the supernatant, i.e., fresh BCJ, which was transferred to sterile bottles and stored at 4℃ until sterilization treatment.

### Sterilization process

Taking fresh BCJ as the control check (CK), TP, HTLT, UHT, HHP and TS were performed respectively. TP and HTLT were carried out using a electric water bath, 50 mL of fresh BCJ was placed in a sterile glass bottle, and kept at 60℃ for 30 min (TP) or 90℃ for 3 min (HTLT); then the BCJ cooled to 20℃ immediately and transferred to 250 mL sterile polyethylene terephthalate (PET) bottles. UHT was performed using an ultra-high temperature instantaneous sterilization system (FT74X, Armfield, Hampshire, UK), where the BCJ was sterilized at 130℃ for 5 s and then filled into PET bottles. Fresh BCJ was subjected to HHP in PET bottles using a HHP-700 hydrostatic pressurization unit (30L-HHP600MPa, Bao Tou KeFa, Inner Mongolia, China) at 25℃ for 500 Mpa and continued for 10 min. TS was performed using an ultrasonic instrument (ATPIO-1000D, Nanjing Xianou, Jiangsu, China); the samples were placed in a double-wall cylindrical vessel connected with a water bath (XODC-0515-II, Nanjing Xianou, Jiangsu, China), at 55℃, 700 W, 20–25 kHz, with a pulse duration of 2 s on and 3 s off, and a duration of 10 min, after which the samples were transferred to PET bottles. All sterilization conditions were set based on preliminary tests to ensure that commercial sterility were met under mild conditions.

### Microbiological Assay

The microbiological assay, including total bacterial content (TBC), Escherichia coli (*E.coli*) and mold and yeast was carried out according to the previously described methods (Lan et al., 2021), and the results were expressed as CFU/mL or MPN/mL in Supplement Table 1. The TBC, *E.coli* and mold and yeast of sterilized BCJ all met the commercial sterility specified in the National Standard of China GB 7101–2022, that was, the five sterilization treatments and conditions described in section 2.3 can ensure the microbiological safety of BCJ.

**Table 1:** Physicochemical properties of sterilized BCJ.

	pH	TA (g/ mL)	TSS (°Brix)	Viscosity (mPa·s)	BI
CK	5.98 ± 0.00 a	382.65 ± 7.28b	5.1 ± 0.1c	2.73 ± 0.01b	3.09 ± 0.03 a
TP	5.82 ± 0.01c	442.41 ± 5.01 a	4.4 ± 0.1 e	2.02 ± 0.03 cd	2.67 ± 0.04c
HTLT	5.83 ± 0.00c	436.63 ± 5.01 a	5.2 ± 0.0b	2.67 ± 0.19b	2.71 ± 0.01c
UHT	5.8 ± 0.00 e	431.81 ± 3.34 a	4.9 ± 0.0 d	2.33 ± 0.59 bc	2.89 ± 0.02b
HHP	5.96 ± 0.01b	385.54 ± 12.04b	5.3 ± 0.1 a	3.37 ± 0.21 a	2.45 ± 0.01 d
TS	5.81 ± 0.01 d	388.43 ± 6.02b	2.6 ± 0.0f	1.57 ± 0.15 d	3.09 ± 0.02 a
CV (%)	1.35%	6.91%	22.40%	25.49%	9.09%

Note: Different letters in the same column represent significant difference (*p* < 0.05).

### Physicochemical characteristics

The pH values were measured by a PHS-3E pH meter (Leici, Shanghai, China). The titratable acid (TA) was determined using the acid-base titration method according to the National Standard of China GB/T 12456–2008, and the results were expressed as g/L. The total soluble solids (TSS) were measured using a PAL-1 digital Abbe Refractometer (ATAGO, Tokyo, Japan), and the results were expressed as °Brix. The viscosity were measured at 30 RPM (Revolutions Per Minute) by rotor 1 of an NDJ-5S rotary viscometer (Jingtian, Jinan, China), and the results were expressed as mPa·s. The browning index (BI) was measured by UV-2800 spectrophotometer (UNICO, Shanghai, China) based on the method of Martins et al. (2021).

### Sensory characteristics

#### Color analysis

The color characteristics were measured in the transmission mode by Ci7600 colorimeter (X-rite, Michigan, USA). The parameters measured included brightness (L*), red-greenness (a*) and yellow-blueness (b*); chroma (C*), hue (h°), and total color difference (ΔE) were automatically calculated by the built-in software.

#### Electronic Nose (E-Nose) assay

The odor profile of BCJ was determined by a PEN 3 electronic nose (Airsense Analytics, Schwerin, Germany) referring to Lan et al. (2021).

#### Sensory evaluation

Sensory evaluation referred to the methods of Ma et al. (2015) with some modification. Thirty well-trained panelists (15 men and 15 women, from 20 to 50 years old) formed the panel to participate in the test. Before the evaluation, the panel received 6 h (6 × 1 h sessions during a week) of training sessions in BCJ evaluation. During the sessions, by comparing to CK, the panel agreed that the sensory attributes of BCJ included color, appearance, aroma, taste and total acceptability and established the hedonic criteria scale (Supplement Table 2). In the formal test, 50 mL BCJ at 25℃ were placed in glass bottles, randomly coded by three digits, and provided to 30 panelists, who rated the five sensory attributes according to Supplement Table 2. All experimental protocols were approved by the Ethics Committee of Northwest A&F University, and the ethics review document was placed at the end of this article.

**Table 2:** Comprehensive quality score and ranking of sterilized BCJ.

sample	Entropy weight-TOPSIS		CRITIC-Topsis		GRA		PCA		average	
	score	rank	score	rank	score	rank	score	rank	score	rank
CK	0.54	4	0.60	4	0.38	4	0.56	5	0.52	4
TP	0.77	1	0.78	1	0.41	2	0.70	2	0.67	1
HTLT	0.70	3	0.71	2	0.39	3	0.61	4	0.60	3
UHT	0.48	5	0.53	5	0.30	5	0.10	6	0.36	6
HHP	0.72	2	0.66	3	0.42	1	0.81	1	0.65	2
TS	0.36	6	0.36	6	0.26	6	0.63	3	0.40	5

### Functional indexes

#### Total polyphenols (TPC), flavonoids (TFC), anthocyanins (TAC) and carotenoids (TCC) content

TPC and TFC were determined using Folin-Ciocalteu method (Dong et al., 2022, Lan et al., 2021), and AlCl_3_ colorimetric method (Ma et al., 2019) and quantified by standard curves. The results were expressed as mg GA equivalents (GAE)/L and mg CA equivalents (CAE)/L, and the standard curves were A_765 nm_ = 0.0045 × TPC + 0.0566 (r^2^ = 0.9990), A_506 nm_ = 0.0017 × TFC + 0.0246 (r^2^ = 0.9992), respectively.

TAC was determined by pH-differential method (Sun et al., 2020), and the results were calculated according to Equation (1) and expressed as mg C3G equivalents (C3GE)/L.

(1) $$TAC=\frac{[{\left(A_{510\;nm}-A_{700\;nm}\right)}_{pH1.0}-{\left(A_{510\;nm}-A_{700\;nm}\right)}_{pH4.5}]\times MW\times DF\times 1000}{\varepsilon \times 1}$$

where MW was the average molecular weight of C3G with a value of 449, DF was the dilution factor, and ε was the extinction coefficient with a value of 29600.

The TCC was carried out according to the method described by de Carvalho et al. (2012), and the results were calculated according to Equation (2) and expressed in mg/L.

(2) $$TCC=\frac{10000\times A_{450\;nm}\times V}{A_{1\;cm}\times V_{0}}$$

where V was the volume of extraction solution, V_0_ was the volume of BCJ for extraction, and A_1 cm_ was the average extinction coefficient of carotenoids with a value of 2500.

#### Antioxidant capacity

According to the method described by Ramírez-Melo et al. (2022), three indexes were used to estimate the antioxidant capacity of BCJ, including DPPH radical scavenging activity (DPPH), ABTS radical cation scavenging activity (ABTS), and ferric reducing antioxidant power (FRAP). The results were quantified through standard curves and expressed as mmol trolox equivalents (TE)/L. The standard curves were A_517 nm_ = -1.3725 × DPPH + 1.7549 (r^2^ = 0.9995), A_593 nm_ = 1.1489 × FRAP + 0.27264 (r^2^ = 0.9990), and A_734 nm_ = -1.6215 × ABTS + 0.8100 (r^2^ = 0.9900), respectively.

### Statistical analysis

The establishment of the mathematical models were carried out in Excel 2020 (Microsoft Office, WA, USA). ANOVA combined with Duncan’s tests, PCA, LDA and HCA were performed by SPSS 23 (IBM SPSS, Chicago, USA). Pearson’s correlation tests and the heatmap were performed by Origin 9.1 (OriginLab, MA, USA). The experiments were performed at least in triplicate and the results were expressed as the mean values ± standard deviation (SD).

## Results and discussions

### Effects of sterilization methods on the physicochemical properties

**Fig. 1:**
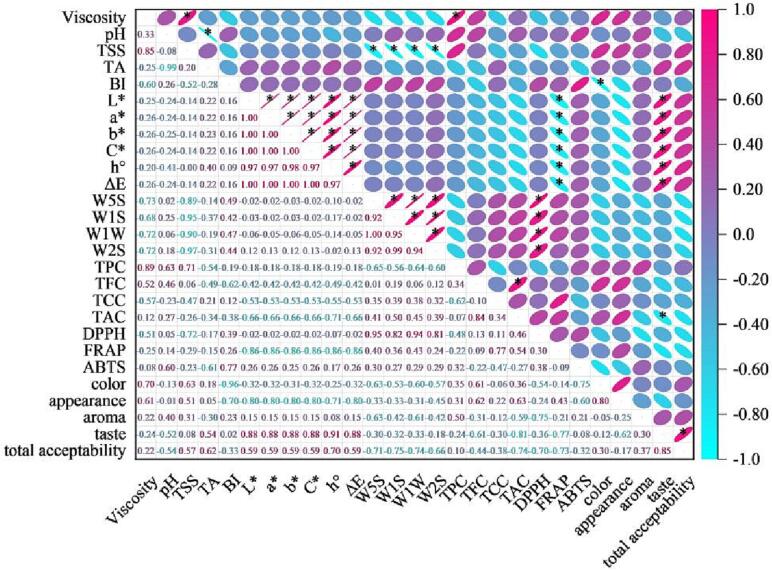
Pearson correlation analysis for all indicators of BCJ. “*” indicates significant correlation (*p* < 0.05).

The pH value of CK was 5.98, and all five sterilization treatments resulted in a significant decrease (*p* < 0.05) (Table 1), which was also found in orange carrot juice (Pokhrel et al., 2019, Umair et al., 2022). This was probably because bonds between hydrogen and acidic molecules were reversible and might be affected by sterilization treatments to release hydrogen ions from acids to the environment (Pokhrel et al., 2019). Similar to pH, TA could also reflect the acidity. The coefficient of variation (CV) of TA was 6.91%, greater than that of pH value (1.32%) (Table 1), indicating that TA varied more among groups and was more suitable as an index of acidity in BCJ. Thermal sterilization caused a significant increase in TA (*p* < 0.05), while HHP and TS had no significant effect (*p* > 0.05). Viscosity was positively correlated with TSS (R_viscocity-TSS_ = 0.85, *p* < 0.05) (Fig. 1), and the variation trend for both was HHP > HTLT > CK > UHT > TP > TS. BI was significantly decreased after sterilization treatments (*p* < 0.05) besides TS, with the most significant decrease after HHP. The reduction of BI after sterilization was also reported in grape juice (Ma et al., 2020) and jujube pulp (Shen, Gou, Zhang, & Wang, 2016), which was mainly due to the inactivation of enzymes related to enzymatic browning during sterilization, thus the enzymatic browning of juice was inhibited (Szczepańska et al., 2020).

### Effects of sterilization methods on the sensory properties

#### Color analysis

**Fig. 2:**
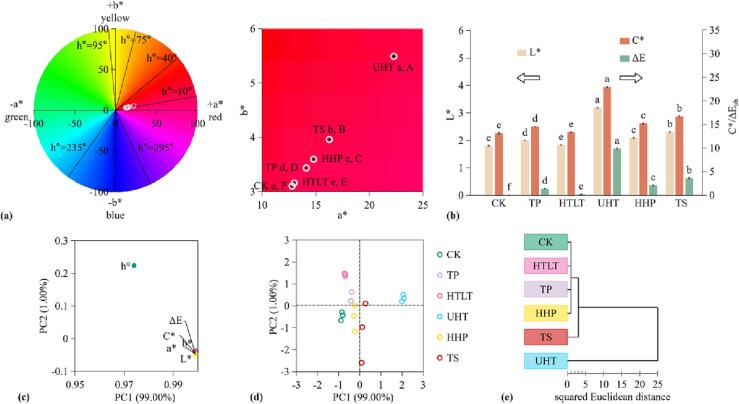
Color characteristics of sterilized BCJ. (a) a*-b* value chromaticity distribution diagram. (b) changes of L* value, C* value and ΔE. (c) PCA loading diagram of each color characteristic. (d) PCA score diagram of different sterilization treatments. (e) HCA clustering diagram. Different lowercase letters in the figure indicate significant differences (*p* < 0.05); different capital letters in (a) indicate significant differences in b* value (*p* < 0.05).

Color is one of the most important sensory attributes of juices, directly affecting consumers’ preference, acceptance and selection of products. All samples were distributed in the positive direction of a* (12.79–22.27), b* (3.10–5.49) and the red zone of h° (13.63–13.86) (Fig. 2(a)). All sterilization treatments significantly increased the redness and yellowness of BCJ (*p* < 0.05) except for HTLT, among which UHT increased the most, and TP increased the least. The L* and C* values represented for brightness and color saturation, respectively. Except for HTLT, the other four sterilization treatments could significantly increased brightness and color saturation of BCJ (*p* < 0.05) (Fig. 2(b)), with the greatest improvement after UHT. The color difference could be distinguished with the naked eye when ΔE > 3 (Cejudo-Bastante et al., 2016). ΔE of the TP and HTLT groups with lower temperatures were<3, and their color difference with CK could not be observed, while that of the UHT group was 9.87, indicating that temperature had a significant effect on BCJ color during the sterilization, and the higher the temperature, the greater the color change. The similar phenomenon was also observed by Yuan et al. (2022), where the ΔE of pomegranate juice after HTST (110℃ 8.6 s) was significantly higher than that of TP (85℃ 30 s). However, some studies also found that low temperature and long time treatment resulted in more drastic color changes in juice compared to high temperature and short time treatment (Wang et al., 2018, You et al., 2018). Therefore, which factor has more significant effects on juice color during thermal sterilization, temperature or time, still need further study. ΔE of the HHP group was 2.09, and the color difference was indistinguishable from CK, which was possibly because HHP did not cause damage to covalent bonds, so that low molecular compounds such as pigments were hardly affected (Ma et al., 2022).

PCA results showed that the variance contribution of PC1 was 99.00%, and the loading values of L*, a*, b*, C*, h° and ΔE in its positive direction were all>0.97 (Fig. 2(c)). The TP, HTLT and HHP points were close to CK (Fig. 2(d)), suggesting that the three treatments did not cause significant changes in the overall color characteristics of BCJ. On the contrary, the TS and UHT points shifted in the positive direction of PC1, implying that TS and UHT changed the overall color characteristics, specifically significantly increasing the brightness, redness, yellowness, saturation, hue and color difference values (*p* < 0.05). This result could also be observed in Fig. 2(e), where UHT and TS were clustered separately while TP, HTLT, HHP and CK were clustered together when the distance < 4.

#### E-nose analysis

**Fig. 3:**
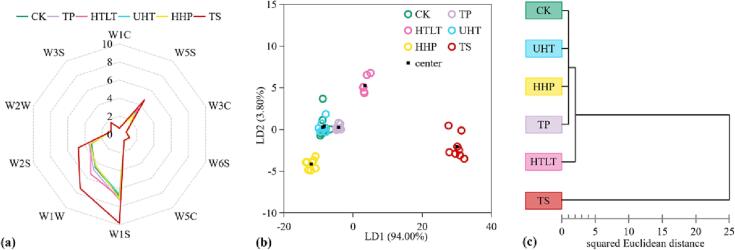
E-nose results of sterilized BCJ. (a) radar chart of E-nose response data. (b) LDA results of e-nose response. (c) HCA clustering diagram.

The sensor types of E-nose that responded to the six groups of BCJ odor were the same, but the response values differed (Fig. 3(a)). W1S showed the strongest response of 6.62–9.82, followed by W1W (4.36–7.37), W2S (3.25–4.78) and W5S (2.79–4.72), indicating that volatiles in BCJ were dominated by methane, sulfur organic compounds, terpenes, alcohols and nitrogen oxides (Supplement Table 3). In Fig. 3(b), the CK, TP and UHT points were distributed closely and even overlapped in some areas, indicating that UHT and TP did not change the overall odor profile of BCJ. This result could also be observed in Fig. 3(c) that CK, TP and UHT were clustered into one class when the distance < 2.

Compared with CK, the HHP group had a negative shift in the direction of LD2 (Fig. 3(b)), but LD2 only explained 3.80% of the variance, indicating that the difference between HHP and CK was small and therefore the two groups could not be separated in HCA (Fig. 3(c)). In addition, both LDA and HCA clearly distinguished the HTLT and TS groups from CK (Fig. 3(b-c)), which meant that the overall odor profile of the two groups changed significantly and could be identified by E-nose. Specifically, the response values of W5S and W1W to HTLT were significantly increased (*p* < 0.05), and the response values of W1S, W1W, W2S and W5S to TS were significantly increased (*p* < 0.05) (Fig. 3(a)). The above findings suggested that TS induced most significant changes in odor profile of BCJ, and similar conclusions were obtained in inulin soursop whey beverage (Guimarães et al., 2019), which was possibly due to the fact that ultrasound promoted the release of glycosidically bound volatiles in the juice through sonochemical effect (Sun et al., 2021).

#### Sensory evaluation

The appearance scores of sterilized BCJ ranged from 17.03 to 17.60 and did not show significant differences (*p* > 0.05) (Figure S1), and the juices all showed a clarified and homogeneous state. Total acceptability was positively correlated with taste score (R_acceptability-taste_ = 0.85, *p* < 0.05) (Fig. 1), indicating that taste was the determinant of total acceptability in BCJ. Therefore, the UHT group with the highest taste score also exhibited the best total acceptability. Moreover, the overall odor profile of the UHT group was basically consistent with CK, thus its aroma score was second only to CK and ranked first among five sterilization groups. However, the color characteristics of the UHT group were significantly different from the other five groups (Fig. 2(a) 2(d)), resulting in lower color score. The taste and total acceptability scores of the TP group were second only to UHT, and its aroma score was lower than that of CK and UHT, ranking second among the five sterilization groups. At the same time, the TP group also obtained a higher color score, second only to HHP among all groups. Hence the TP group exhibited a relatively pleasant sensory evaluation profile.

The color score was negatively correlated with BI (R_color-BI_ = -0.96, *p* < 0.05) (Fig. 1), suggesting that the degree of browning was an important factor affecting consumers’ preference for the color of BCJ, and all five sterilization treatments significantly reduced the browning, improving its color characteristics. Among the six groups, the BI of the HHP group was significantly the lowest (*p* < 0.05) (Table 1), giving it the highest color score (Figure S1). However, the HHP group had poorer taste score and thus ranked third in the total acceptability score among all sterilization groups, and was not significantly different from CK (*p* < 0.05), that was, HHP could better maintain the sensory evaluation profile of fresh BCJ, which was also confirmed in many juice matrices (Inada et al., 2018, Picouet et al., 2015, Zhao et al., 2021). The TS group had lower scores in all five attributes (Figure S1), implying that TS caused severe deterioration in the sensory quality of BCJ. However, TS under suitable conditions could improve the sensory quality of hog plum juice (Oladunjoye, Adeboyejo, Okekunbi, & Aderibigbe, 2021) and blood fruit juice (Raju, & Deka, 2018). Accordingly, operating parameters such as temperature and ultrasonic power were key factors to determine the sensory quality of thermalsonicated juice. Besides, it should not be overlooked that even the same sterilization method and conditions could produce different effects on different juice matrices.

### Effects of sterilization methods on the functional indexes

#### TPC, TFC, TAC and TCC

**Fig. 4:**
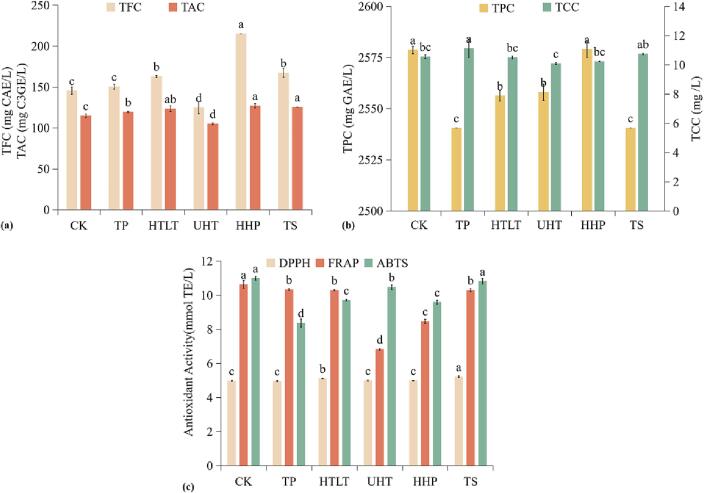
Functional indexes of sterilized BCJ. (a) the changes of TFC and TAC. (b) the changes of TPC and TCC. (c) the changes of DPPH, FRAP and ABTS antioxidant activity. Different lowercase letters in the figure indicate significant differences (*p* < 0.05).

TPC, TFC and TAC of CK were 2578.68 mg GAE/L, 145.52 mg CAE/L and 114.87 mg C3GE/L, respectively (Fig. 4(a-b)). All sterilization except HHP led to a significant decrease in TPC (*p* < 0.05), but the decrease rate was only 0.80–1.48%, suggesting that sterilization treatment did not cause a large loss of polyphenols in BCJ. This phenomenon was probably because the polyphenols in black carrot were dominated by anthocyanins and phenolic acids (Smeriglio et al., 2018), and the high stability of acylated anthocyanins as well as the intermolecular or intramolecular co-pigmentation of phenolic acids contributed greatly to the stability of black carrot polyphenols (Gras, Bogner, Carle, & Schweiggert, 2016).

Compared with CK, TFC was significantly increased by 47.79% and 15.02%, and TAC by 10.65% and 9.28% in the HHP and TS groups (*p* < 0.05), respectively, ranking the top two among all groups (Fig. 4(a)). HHP disrupted the integrity of cell walls and membranes through pressure (Inada et al., 2018), while TS led to the disruption of cell walls and vacuoles of plant tissues as well as cavitational collapse in the surrounding colloidal particles, both contributing to improve the extractability of phenolic compounds in food matrices (Ma et al., 2020). In the thermal sterilization groups, HTLT significantly increased TFC and TAC (*p* < 0.05). TP significantly increased TAC (*p* < 0.05) and had no significant effect on TFC (*p* > 0.05). However, UHT significantly decreased TFC and TAC by 14.16% and 8.55% compared with CK, respectively (*p* < 0.05). This might be because moderate temperature was beneficial to the extractability of flavonoids and anthocyanins in juice matric, while excessive temperature led to the degradation of both.

The TCC of CK was 10.57 mg/L, which was significantly increased by TP (*p* < 0.05), while had no significant changes after other sterilization treatments (*p* > 0.05) (Fig. 4(b)). Sterilization treatments could facilitate the release of carotenoids from the food matrix by disrupting the structure of plastids, promoting the disintegration of crystalline carotenoids and breaking the covalent bonds of the protein-pigment complexes. However, higher temperatures during sterilization could promote the oxidative degradation of carotenoids, so the impact of sterilization on TCC was jointly determined by the two aspects (Ngamwonglumlert, Devahastin, Chiewchan, & Raghavan, 2020). Furthermore, the CV of TCC was only 3.50%, with little variance among groups, implying that carotenoids in BCJ were basically stable.

#### Antioxidant activity

The FRAP, DPPH and ABTS of CK were 10.63, 4.98 and 11.00 mmol TE/L, respectively (Fig. 4(c)), which were significantly higher than those of other colored carrots (Singh et al., 2018). All sterilization treatments resulted in a significant reduction in FRAP (*p* < 0.05), with the highest reduction rate in UHT at 35.76%. TS and HTLT resulted in a significant enhancement of DPPH (*p* < 0.05), while other treatments did not have significant effects (*p* > 0.05). All sterilization groups except TS resulted in a significant decrease (*p* < 0.05) in ABTS, with the highest decrease rate in the TP group of 23.83%. Combining the three indexes, TS was the most conducive to the enhancement or retention of antioxidant activity in BCJ, and the same conclusion was drawn in grape (Ma et al., 2020) and hog plum (Oladunjoye et al., 2021) juice.

### Comprehensive quality of sterilized BCJ quantified by PCA, TOPSIS and GRA models

#### Selection of evaluation indexes and construction of matrix

28 indexes including physicochemical properties, color properties, E-nose response values and functional indexes were measured. Since the physicochemical properties, color properties and e-nose response values cannot directly reflect the BCJ quality, and some of them did not vary significantly among groups, the above indexes were screened according to CV (CV > 10%) or their correlation with sensory evaluation (*p* < 0.05). Under these conditions, 12 indexes including viscosity, TSS, BI, L*, a*、b*, C*, ΔE, W5S, W1S, W1W and W2S were screened out. Functional indexes directly reflected the quality, and the larger the value, the better the quality, which were all used for the next analysis. Thus 19 indexes including TPC, TFC, TAC, TCC, DPPH, FRAP, ABTS, BI, viscosity, TSS, W5S, W1S, W1W, W2S, L*, a*, b*, C* and ΔE were screened to construct the evaluation models for the BCJ comprehensive quality. The BCJ quality evaluation matrix was constructed based on 19 indexes:

$$X=\left[\begin{array}{c} x_{11}x_{12}\cdots .x_{119}\\ x_{21}x_{22}\cdots x_{219}\\ \vdots \vdots x_{\mathrm{ij}}\vdots \\ x_{61}x_{62}\cdots x_{619} \end{array}\right]$$

where i ranged from 1 to 6 and j ranged from 1 to 19, indicating the measured value of the jth index of the ith sterilization group, and the specific values were shown in Supplement Table 4.

According to the evaluation effect, the indexes could be divided into maximum (the larger the value, the better the quality), minimum (the smaller the value, the better the quality), intermediate (the closer the value was to a certain value, the better the quality) and interval (the quality was best when the value was in a certain interval) indexes. The minimum, intermediate and interval indexes needed to be processed to maximum indexes before modeling. Consequently, it was necessary to determine the category to which the 19 indexes belong first.

Obviously, TPC, TFC, TAC, TCC, DPPH, FRAP and ABTS were maximum indexes and did not need to be positive processed, i.e. $y_{i}=x_{i}$. E-nose response values, physicochemical and color properties cannot directly determine the BCJ quality, but were closely related to sensory quality and therefore classified based on their correlation with sensory evaluation. BI was significantly negatively correlated with color score (*p* < 0.05) (Fig. 1), and the smaller the BI, the higher the color preference, which belonged to minimum index, and was processed to the maximum index according to Equation (3):

(3) $$y_{i}=max-x_{i}$$

where max was the largest BI among the six groups, i.e., the BI of CK, $x_{i}$ was the BI of each group, and $y_{i}$ was the BI of each group after positive processing. W5S, W1S, W1W, and W2S were related to the aroma score, but there was no significant correlation. CK had the highest aroma score with response values of 2.95, 6.85, 4.53 and 3.25 for the four sensors, illustrating that the closer the response values were to CK, the better the aroma preference, thus the E-nose response values belonged to intermediate index, and $x_{\mathrm{best}}=x_{\mathrm{CK}}$. Similarly, viscosity and TSS were also intermediate indexes, and the UHT group with the highest taste score was the optimal sample, $x_{\mathrm{best}}=x_{\mathrm{UHT}}$. Color properties L*, a*, b*, C* and ΔE were also intermediate indexes, and the HHP group with the highest color score was the optimal sample, $x_{\mathrm{best}}=x_{\mathrm{HHP}}$. Intermediate indexes were processed to maximum indexes according to Equation (4):

(4) $$y_{i}=1-\frac{x_{i}-x_{\mathrm{best}}}{max\left\{\left|x_{i}-x_{\mathrm{best}}\right|\right\}}$$

where $\max\{\left|x_{i}-x_{\mathrm{best}}\right|\}$ referred to the max value of $\left|x_{i}-x_{\mathrm{best}}\right|$, and *i* ranged from 1 to 6.

$$Y=\left[\begin{array}{c} y_{11}y_{12}\cdots .y_{119}\\ y_{21}y_{22}\cdots y_{219}\\ \vdots \vdots y_{\mathrm{ij}}\vdots \\ y_{61}y_{62}\cdots y_{619} \end{array}\right]$$

In summary, all indexes were positive processed, and the positive BCJ quality evaluation matrix Y was constructed. The matrix Y was standardized according to Equation (5) to eliminate the effects caused by different index magnitudes, and the standardized BCJ quality evaluation matrix Z was obtained and subsequently analyzed.

(5) $$z_{\mathrm{ij}}=\frac{y_{\mathrm{ij}}}{\sum_{i=1}^{6}\sqrt{y_{\mathrm{ij}}^{2}}}$$

$$Z=\left[\begin{array}{c} z_{11}z_{12}\cdots .z_{119}\\ z_{21}z_{22}\cdots z_{219}\\ \vdots \vdots z_{\mathrm{ij}}\vdots \\ z_{61}z_{62}\cdots z_{619} \end{array}\right]$$

#### Construction of comprehensive evaluation model

Based on the Z matrix, the Entropy weight-TOPSIS, CRITIC-TOPSIS, GRA and PCA models were developed reffering to the descriptions of Liang et al., 2019, Liu et al., 2022, Wang *et al.* (2021a) and Wang *et al*. (2021b), and the weight of each index (Supplement Table 5) and the comprehensive score of BCJ were calculated (Table 2). Different mathematical models had different methods of assigning weights to indexes. For example, the Entropy weight method considered that the dispersion of indexes was positively correlated with their weights. As a result, sensory and physicochemical indexes were more discrete than functional properties and thus had higher weight values in Entropy weight-TOPSIS model (Supplement Table 5), and the importance of functional properties were weakened. In order to prevent a single model from assigning too much weight to one type of indexes, reducing the accuracy and comprehensiveness, the average scores of the four models were calculated to evaluate the comprehensive quality of BCJ (Table 2).

Compared with CK, the mean score of TS and UHT groups decreased (Table 2), implying the comprehensive quality deteriorated. The TS group ranked 5th on average and showed poor comprehensive quality. It had the worst E-nose response values, which reflected a severe deterioration in odor profile. Moreover, its TSS differed the most from the optimal value UHT. Nevertheless, the TS group had significantly higher TFC and TAC (*p* < 0.05) and the strongest antioxidant activity among the sterilization groups. Hence the hurdle sterilization TS was a nutritional high-value processing method for BCJ, but had significant adverse effects on sensory and physicochemical properties. The UHT group had the worst comprehensive quality, with a mean score reducing by 30.77% compared to CK due to its worst color and functional properties. The mean scores of the TP, HTLT and HHP groups were higher than that of CK, implying that the three treatments improved the comprehensive quality of BCJ. The TP group showed the best comprehensive quality, with a mean score of 0.67, an improvement of 28.85% compared to CK, and both sensory and functional indexes were improved or retained, which indicated that TP treatment was the most appropriate sterilization method for BCJ. The HHP group ranked 2ed, with a mean comprehensive quality score of 0.65, whose superiority was the E-nose response values close to those of CK, the significantly highest TFC and TPC (*p* < 0.05), and the most superior color properties.

Sterilization was the key step in juice processing and had a decisive influence on its safety and quality. Thermal sterilization was commonly considered to deteriorate the sensory and nutritional qualities, however, appropriate temperature and time did not negatively affect the juice quality (Dereli et al., 2015, Wurlitzer et al., 2019). Non-thermal sterilization was believed to be more advantageous than thermal sterilization, but they cannot always achieve significantly superior effects than the latter (Picouet et al., 2015, You et al., 2018). In addition, the cost of non-thermal sterilization was much higher, thus a comprehensive consideration should be made when selecting sterilization methods. To sum up, whether thermal or non-thermal sterilization, their influence on microorganisms, enzyme and the comprehensive quality of juice were closely related to the processing parameters, microbial species and the juice matrices themselves. In the future research and production, the most suitable sterilization methods and the best process parameters should be explored for different types of juice matrices, rather than blindly pursuing non-thermal sterilization technologies.

## Conclusion

The comprehensive quality of BCJ sterilized through three thermal (TP, HTLT, UHT), one non-thermal (HHP) and one hurdle (TS) methods were investigated and quantified based on PCA, TOPSIS and GRA models for the first time. TP, HTLT and HHP did not change the color characteristics of BCJ, while UHT and TS significantly increased its brightness, redness, yellowness, saturation, and hue. UHT and TP essentially did not alter the odor profile, whereas TS induced the most drastic changes. Taste was the determinant of total acceptability in BCJ. Both UHT and TP groups exhibited a pleasant sensory profile, while the sensory of TS group deteriorated seriously. Polyphenols and carotenoids were stable in BCJ, whose contents were basically unaffected by sterilization. HHP and TS were most beneficial to increase flavonoids and anthocyanins contents in BCJ, while UHT significantly reduced them. TS was most conducive for improving the antioxidant activity of BCJ. According to the models, TP and HHP, especially TP made the greatest improvement in comprehensive quality, while UHT caused the most severe deterioration. Therefore, suitable sterilization methods should be explored for specific juice matrices, rather than blindly pursuing non-thermal sterilization. This study determined the most favorable sterilization method to improve the comprehensive quality of BCJ, and provided a more feasible and scientific solution for the screening of high quality processing methods.

## Funding

This work was supported by the National Nature Science Foundation Project (31801560), the Innovation Capacity Support Plan of Shaanxi Province (2022NY-039, 2022ZDLNY04-04, 2023-YBNY-176, 2020-TD-47).

## CRediT authorship contribution statement

**Shihan Bao:** Methodology, Investigation, Data curation, Visualization, Writing – original draft. **Dingze Yin:** Methodology, Investigation. **Qinyu Zhao:** Methodology, Investigation, Data curation. **Yuan Zhou:** Methodology, Investigation, Data curation. **Yayun Hu:** Methodology, Investigation, Data curation. **Xiangyu Sun:** Conceptualization, Writing – review & editing. **Xuebo Liu:** Methodology, Investigation, Data curation. **Tingting Ma:** Conceptualization, Methodology, Writing – review & editing.

## Declaration of Competing Interest

The authors declare that they have no known competing financial interests or personal relationships that could have appeared to influence the work reported in this paper.

## Data Availability

Data will be made available on request.
