# Supplementary material for: Comprehensive evaluation of the effect of five sterilization methods on the quality of black carrot juice based on PCA, TOPSIS and GRA models
Source: Food Chem X. 2023 Feb 21;17:100604. doi: 10.1016/j.fochx.2023.100604 (PMC10039260; doi:10.1016/j.fochx.2023.100604)

**Supplement Table 1. Microbial inactivation of BCJ by different sterilization treatments**

|  | TBC  (CFU/mL) | *E.coli*  (MPN/mL) | Mold and yeast (CFU/mL) |
| --- | --- | --- | --- |
| CK | 275.00±14.14 a | 0.64±0.40 | 1.17±1.18 |
| TP | 1.00±1.73 b | nd | nd |
| HTLT | nd | nd | nd |
| UHT | 16.83±23.33 b | nd | nd |
| HHP | 0.67±0.94 b | nd | nd |
| TS | 7.95±2.89 b | nd | nd |

Note: Different letters represent significant difference (*p*<0.05) from each other in the same column. “nd” means not detected.

**Supplement Table 2. Sensory hedonic criteria scale**

| score | color | appearance | aroma | taste | acceptability |
| --- | --- | --- | --- | --- | --- |
| 16~20 | Dark purple, good luster. | Clarified, transparent, no precipitation, no stratification. | Strong aroma, no bad odor such as steaming smell. | Round mouthfeel, suitable acidity and sweetness, no astringency, bitter and other bad taste. | Very pleased to accept. |
| 11~15 | Darker or lighter purple, luster. | Slightly turbid, slight precipitation, stratification. | Average aroma, no bad odor. | Pleasant mouthfeel, slightly astringent, no bitterness, no off-flavor. | Basically acceptable. |
| 6~10 | Brown or almost colorless, poor luster. | More turbid, with a small amount of precipitation or obvious stratification. | Lighter aroma with slight bad odor. | Thin mouthfeel, slightly astringent, no bitterness, slightly off-flavored. | Basically unacceptable. |
| 1~5 | Brown or colorless, lusterless. | Severe turbidity or more precipitation, with obvious stratification. | Basically no aroma, with obvious bad odor such as steaming smell. | Thin mouthfeel, heavy astringency, bitterness and off flavors. | Totally unacceptable. |

**Supplement Table 3. Performance of PEN-3 E-nose sensors**

| Sensor | Performance |
| --- | --- |
| W1C | Benzene and aromatic compounds |
| W5S | Broad-range sensitivity, very sensitive to nitrogen oxides |
| W3C | Ammonia, sensitive to aromatic compounds |
| W6S | Mainly hydrogen, selectively |
| W5C | Alkane, aromatic compounds |
| W1S | Sensitive to methane, broad range. |
| W1W | Sensitive to many sulfur organic compounds and terpenes. |
| W2S | Alcohol, sensitive to aromatic compounds with broad range, similar to No. 6. |
| W2W | Aromatic compounds and sulfur organic compounds |
| W3S | Reacts at high concentrations, very sensitive to several compounds |

**Supplement Table 4. Quality evaluation matrix of BCJ**

| i/Sample | | j/Quality index | | | | | | | | | | | | | | | | | | |
| --- | --- | --- | --- | --- | --- | --- | --- | --- | --- | --- | --- | --- | --- | --- | --- | --- | --- | --- | --- | --- |
|  |  | 1 | 2 | 3 | 4 | 5 | 6 | 7 | 8 | 9 | 10 | 11 | 12 | 13 | 14 | 15 | 16 | 17 | 18 | 19 |
|  |  | TPC | TFC | TCC | TAC | DPPH | FRAP | ABTS | BI | Viscosity | TSS | W5S | W1S | W1W | W2S | L* | a* | b* | C* | ΔE |
| 1 | CK | 2578.68 | 145.52 | 10.57 | 114.87 | 4.98 | 10.63 | 11.00 | 3.09 | 2.73 | 5.1 | 2.95 | 6.85 | 4.53 | 3.25 | 1.80 | 12.79 | 3.10 | 13.16 | 0.00 |
| 2 | TP | 2540.53 | 150.52 | 11.13 | 119.48 | 4.96 | 10.33 | 8.38 | 2.67 | 2.02 | 4.4 | 3.12 | 7.26 | 4.84 | 3.51 | 1.99 | 14.11 | 3.44 | 14.52 | 1.38 |
| 3 | HTLT | 2556.32 | 162.93 | 10.51 | 123.40 | 5.12 | 10.30 | 9.71 | 2.71 | 2.67 | 5.2 | 3.57 | 6.97 | 5.40 | 3.36 | 1.83 | 12.99 | 3.16 | 13.37 | 0.21 |
| 4 | UHT | 2558.07 | 124.92 | 10.10 | 105.05 | 5.00 | 6.83 | 10.48 | 2.89 | 2.33 | 4.9 | 2.97 | 6.63 | 4.45 | 3.40 | 3.19 | 22.27 | 5.49 | 22.94 | 9.87 |
| 5 | HHP | 2579.12 | 215.06 | 10.24 | 127.10 | 4.99 | 8.47 | 9.59 | 2.45 | 3.37 | 5.3 | 2.79 | 7.08 | 4.36 | 3.41 | 2.09 | 14.79 | 3.60 | 15.22 | 2.09 |
| 6 | TS | 2540.53 | 167.37 | 10.75 | 125.53 | 5.22 | 10.30 | 10.82 | 3.09 | 1.57 | 2.6 | 4.72 | 9.82 | 7.37 | 4.78 | 2.30 | 16.25 | 3.96 | 16.73 | 3.62 |

**Supplement Table 5. Weight of quality indexes under different models**

| Qualities  indexes | Entropy  weight | CRITIC | GRA | PCA | | | |
| --- | --- | --- | --- | --- | --- | --- | --- |
|  |  |  |  | PC1 | PC2 | PC3 | PC4 |
| TPC | 0.000 | 0.001 | 0.053 | -0.088 | 0.238 | -0.401 | 0.404 |
| TFC | 0.005 | 0.027 | 0.052 | 0.219 | 0.191 | -0.372 | -0.165 |
| TCC | 0.000 | 0.006 | 0.054 | 0.19 | -0.010 | 0.530 | 0.045 |
| TAC | 0.001 | 0.01 | 0.053 | 0.311 | 0.088 | -0.125 | -0.091 |
| DPPH | 0.000 | 0.004 | 0.053 | 0.178 | -0.300 | -0.122 | -0.042 |
| FRAP | 0.004 | 0.024 | 0.055 | 0.227 | 0.015 | 0.320 | 0.426 |
| ABTS | 0.002 | 0.022 | 0.053 | -0.063 | -0.241 | -0.335 | 0.512 |
| BI | 0.181 | 0.106 | 0.050 | 0.036 | 0.313 | -0.091 | -0.524 |
| Viscosity | 0.095 | 0.127 | 0.053 | -0.255 | -0.093 | 0.374 | 0.013 |
| TSS | 0.07 | 0.077 | 0.051 | -0.24 | 0.275 | 0.045 | 0.045 |
| W5S | 0.073 | 0.078 | 0.051 | -0.221 | 0.299 | 0.058 | 0.078 |
| W1S | 0.069 | 0.071 | 0.052 | -0.197 | 0.319 | 0.060 | 0.088 |
| W1W | 0.072 | 0.076 | 0.051 | -0.215 | 0.311 | 0.045 | 0.075 |
| W2S | 0.070 | 0.071 | 0.052 | -0.200 | 0.315 | 0.054 | 0.112 |
| L* | 0.071 | 0.060 | 0.053 | 0.292 | 0.19 | 0.047 | 0.097 |
| a* | 0.072 | 0.060 | 0.053 | 0.292 | 0.191 | 0.045 | 0.094 |
| b* | 0.071 | 0.060 | 0.053 | 0.292 | 0.190 | 0.049 | 0.099 |
| C* | 0.071 | 0.060 | 0.053 | 0.292 | 0.191 | 0.045 | 0.094 |
| ΔE | 0.071 | 0.060 | 0.053 | 0.292 | 0.191 | 0.045 | 0.094 |


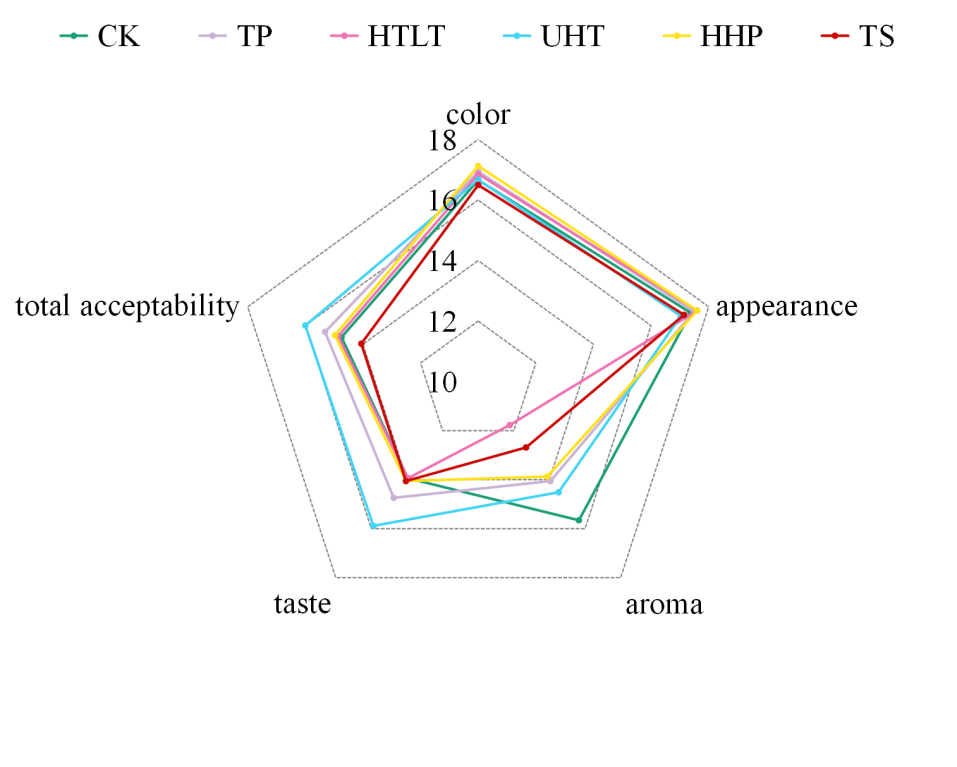


**Figure. S1** Sensory evaluation results of sterilized BCJ.

**Appendice**


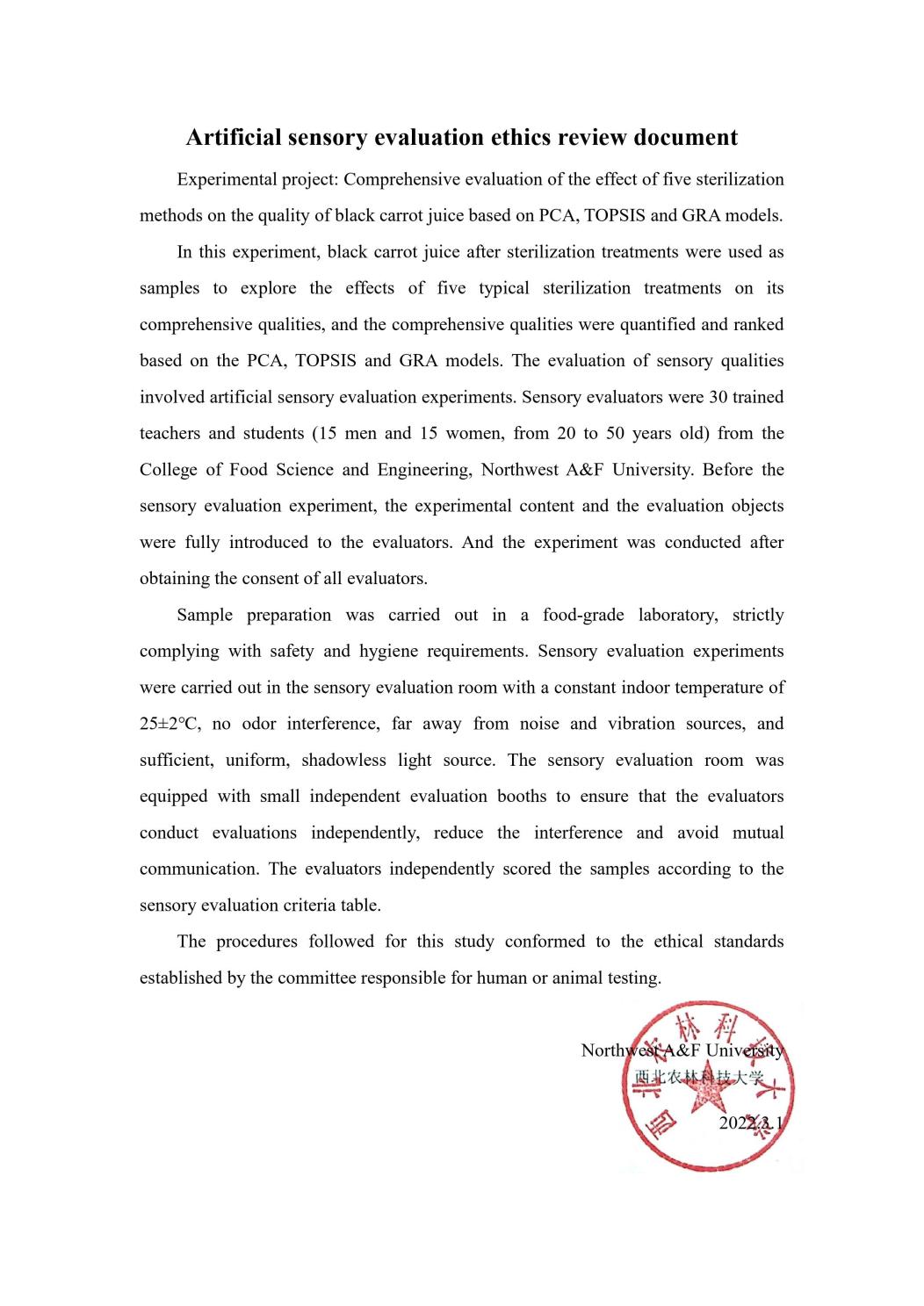

Supplement: Supplementary data 1 [file mmc1.docx]
